# NF-κB in control of regulatory T cell development, identity, and function

**DOI:** 10.1007/s00109-022-02215-1

**Published:** 2022-06-08

**Authors:** Nadine Hövelmeyer, Marc Schmidt-Supprian, Caspar Ohnmacht

**Affiliations:** 1 Institute for Molecular Medicine, University Medical Center of the Johannes Gutenberg-University Mainz, Mainz, Germany; 2 Germany Research Center for Immunotherapy (FZI), University Medical Center of the Johannes Gutenberg-University Mainz, Mainz, Germany; 3 Institute for Experimental Hematology, Center for Translational Cancer Research (TranslaTUM), School of Medicine, Technical University Munich, Munich, Germany; 4 German Cancer Consortium (DKTK) and German Cancer Research Center (DKFZ), 69120 Heidelberg, Germany; 5 Center for Allergy and Environment (ZAUM), Technical University and Helmholtz Center Munich, Munich, Germany

**Keywords:** NF-kappaB, Treg cells, Foxp3, c-Rel, RelA, p100/p52, p105/p50, Bcl-3, Iκbζ, IκB_NS_

## Abstract

Regulatory T cells (Treg cells) act as a major rheostat regulating the strength of immune responses, enabling tolerance of harmless foreign antigens, and preventing the development of pathogenic immune responses in various disease settings such as cancer and autoimmunity. Treg cells are present in all lymphoid and non-lymphoid tissues, and the latter often fulfill important tasks required for the physiology of their host organ. The activation of NF-κB transcription factors is a central pathway for the reprogramming of gene expression in response to inflammatory but also homeostatic cues. Genetic mouse models have revealed essential functions for NF-κB transcription factors in modulating Treg development and function, with some of these mechanistic insights confirmed by recent studies analyzing Treg cells from patients harboring point mutations in the genes encoding NF-κB proteins. Molecular insights into the NF-κB pathway in Treg cells hold substantial promise for novel therapeutic strategies to manipulate dysfunctional or inadequate cell numbers of immunosuppressive Treg cells in autoimmunity or cancer. Here, we provide an overview of the manifold roles that NF-κB factors exert in Treg cells.

## Introduction

A fundamental characteristic of the mammalian immune system is the discrimination between self and non-self, preventing autoimmunity while enabling protective immune responses. Regulatory T cells (Treg) characterized by expression of FOXP3 play a pivotal role in all aspects of the immune response and control immune homeostasis. Indeed, their capacity to suppress the activity of effector immune cells enables them to regulate the duration and intensity of the immune response and to prevent autoimmunity. This was first evidenced in mice lacking the *Foxp3* gene, which resulted in massive systemic autoimmune inflammation [1] and has also been observed in humans with loss-of-function mutations in the *FOXP3* gene, which causes immune dysregulation, polyendocrinopathy, enteropathy, and X-linked (IPEX) syndrome [2].

FOXP3-expressing Treg cells first arise during normal thymic T cell differentiation; these thymic Treg cells represent the majority of all Treg cells in most secondary lymphoid organs and also in the majority of peripheral organs. However, exposure to harmless foreign antigens from diet or commensal microorganisms, particularly at barrier sites such as the lung or gut, requires additional Treg cell differentiation capacity in the periphery to avoid T cell activation. Naïve CD4^+^ FOXP3^−^ T cells that recognize cognate antigens derived from food or microbiota, presented by conventional dendritic cells in draining lymphoid tissues, are able to differentiate into mature and functional CD4^+^FOXP3^+^ Treg cells. In contrast to thymic Treg cells, which typically arise from self-reactive thymocytes, these extrathymically differentiated Treg cells (pTreg) lack expression of the Ikaros family member Helios (*Ikzf2*) and the cell-surface marker Neuropilin-1 and instead express the transcription factor ROR(γt) [3, 4]. Mice lacking pTreg cells develop inflammation and dysbiosis in the lung and gut [5, 6], underlining the importance of this subset for maintaining tolerance at mucosal sites.

The two fundamentally different pathways of Treg development at two distinct locations, within and outside of the thymus, raise the possibility that the mechanisms ensuring their differentiation and lineage stability also differ. Genetic loss of function studies in the mouse demonstrated that both differentiation and lineage stability of Treg cells strongly depended on signals leading to NF-κB activation as well as on individual NF-κB proteins [7–17] (see Box 1). In the following, we focus our discussion on core NF-κB transcription factors and signaling proteins, whose main functions relate to NF-κB activation and omit dissecting the roles of further upstream located mediators with many prominent additional roles. NF-κB family members are able to bind to specific conserved non-coding sequence (CNS 1–3) elements within introns near the *Foxp3* promoter and thereby regulate different aspects of Treg cell development, survival, and function. While the CNS1 region controls predominantly Treg cell differentiation in the periphery [5], CNS2 ensures lineage stability upon cell division, and CNS3 is critical for Treg development in the thymus [18]. Recently, loss-of-function and gain-of-function approaches targeting members of the NF-κB pathway are helping to identify specific roles for different components of this signaling pathway in each Treg subset. The identification of Treg cell–specific roles of NF-κB transcription factors holds substantial promise for selective targeting of Treg cells and their subsets for future cell-based therapies or for directly target Treg cells in clinical settings for the treatment of (auto)immune diseases.

In this review, we summarize biological insights from Treg-specific loss-of-function and gain-of-function mouse models of specific members of the NF-κB pathway, which most insights into the role of NF-κB for Treg function have been gained from. We begin with a discussion of studies targeting canonical NF-κB transcription factors, before reviewing genetic mouse models of alternative NF-κB transcription factors as well as the role of the atypical NF-κB members. We conclude with insights into Treg cell differentiation from human patients.

## Canonical NF-κB transcription factors and Treg cell function

Canonical NF-κB signaling is necessary for proper Treg cell development in the thymus and for maintaining the Treg cell population in the periphery, as evidenced by studies disrupting the expression of IκB-kinase β (IKKβ), a central element of the canonical NF-κB signaling pathway that mediates the development and activation of immune cells. IKKβ ablation in thymocytes prevented the development of Treg cells [19]. In line with this finding, expression of a IκB super-repressor inhibited thymic Treg cell development in mixed bone marrow chimeras, which demonstrated the cell-intrinsic requirement for NF-κB activation [20]. Mice lacking IKKβ specifically in Treg cells developed a spontaneous full-scurfy-like autoimmune syndrome due to the absence of peripheral Treg cells. In these mice, Treg cells matured in the thymus, because the IKKβ gene was excised late during their development, and IKKβ-deficient Treg cells died after release from the thymus. The few Treg cells present in the mice were functional, as they could suppress effector T cells, which they did somewhat more potently than wild-type Treg cells [21].

To date, most of the information about specific peripheral NF-κB protein function and Treg cells has emerged from the analysis of mice with conditional ablation of c-Rel and/or RelA either in T cells or specifically in FOXP3^+^ Treg cells. The latter strategy is in general much more insightful to specifically study the consequences of NF-κB-related Treg cell defects because conventional T cell activity partially relies on the same pathways.

### C-Rel

**Fig. 1:**
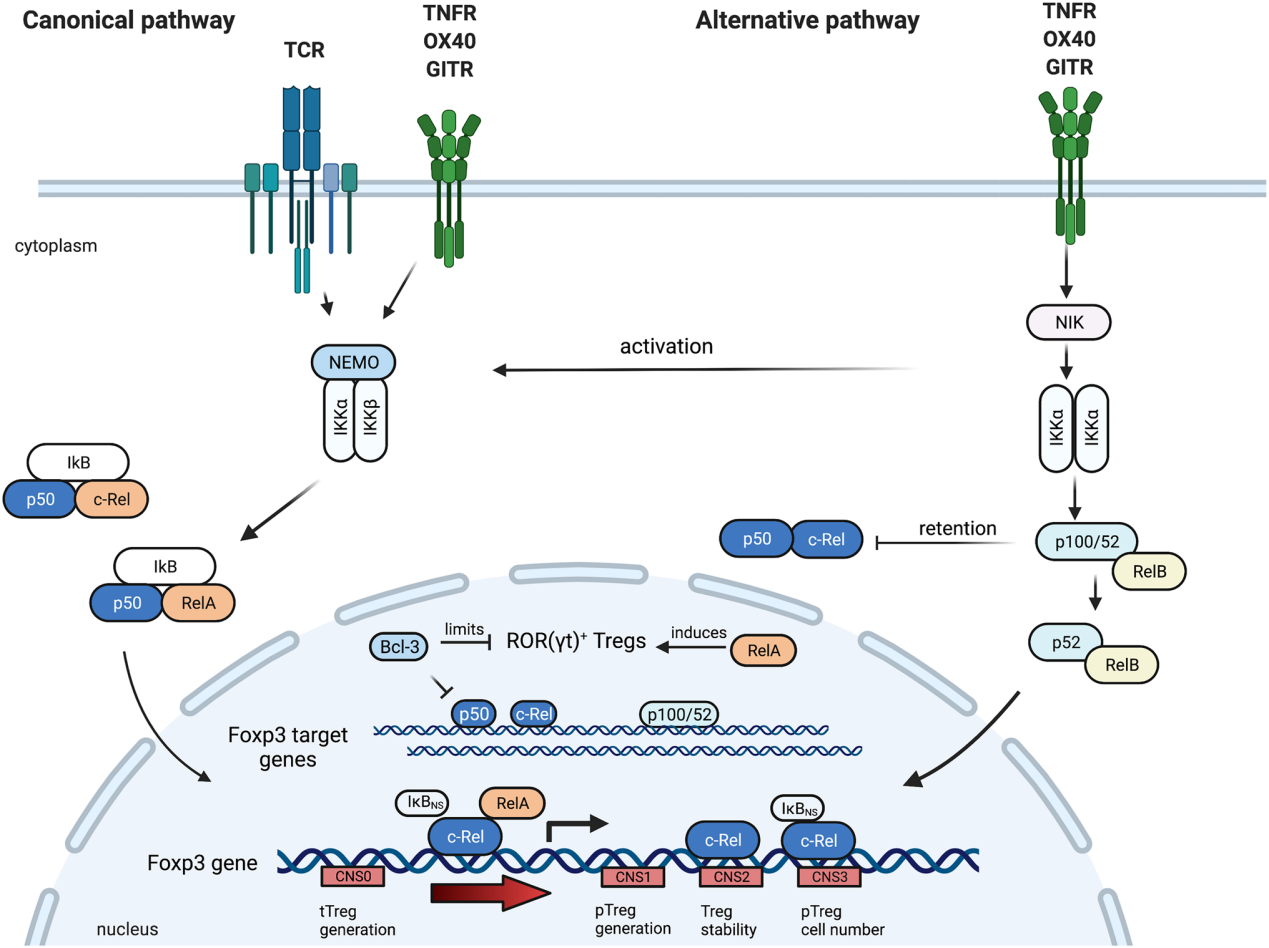
Simplified overview of NF-κB signaling in Treg cells. For the canonical NF-κB pathway (left), T cell receptor (TCR) signaling or signaling via TNF receptor family members (TNF, OX40, GITR) via IκB-kinase β (IKKβ) results in the nuclear translocation of c-Rel and RelA whereby both Foxp3 expression, regulatory elements next to the Foxp3 promotor (CNS0-3) and Foxp3 target gene expression, can be regulated depending on context and Treg cell status/subset. The non-canonical NF-κB pathway (right) equally activated by TNF receptor family members signal via NIK and IKKα has a less pronounced effect on Treg cells but crosstalk with the canonical NF-κB pathway. Atypical IκB members such as Bcl-3 or IκB_NS_ regulate specific Treg cell subsets or certain Treg cell features either directly or via interfering with canonical NF-κB family members

C-Rel employs multiple mechanisms to promote the differentiation as well as thymic and peripheral functions of Treg cells in mice [10, 22, 23]. Indeed, c-Rel can currently be considered as the NF-κB members with the most profound effects on Treg cell biology (see Fig. 1). One reason for this observation is most likely the direct role of c-Rel in regulating FOXP3 expression during Treg cell differentiation. Strong c-Rel expression is a direct consequence of TCR signaling because a comparison of transcriptomes from in vivo–activated T cells and thymocytes with anti-CD3 in vitro–activated thymocytes identified c-Rel as one of the strongest TCR-induced immediate early genes [24]. In line with this observation, enhanced c-Rel expression in Treg cells is strongly dependent on continuous TCR signals [25]. Interestingly, c-Rel-deficient mice do not develop lymphoproliferative or autoimmune disease even though they have only 15% of normal Treg cell numbers, most likely due to general immune deficiency [23, 26]. The deficit in c-Rel-deficient Treg cells manifests already in thymic Treg differentiation and is primarily of quantitative and not qualitative nature [22]. Thus, c-Rel may serve as a switch for developing Treg cells, and it will be interesting to study whether the few developing c-Rel-deficient Treg cells have a skewed TCR repertoire with a distinct affinity for MHC:peptide complexes. At the molecular level, c-Rel may synergize with p65, NFAT, Smad, and CREB, all of which have been show to contribute FOXP3 expression in developing thymic Tregs [10]. Ablation of c-Rel late during Treg cell development does not affect peripheral Treg cell numbers, showing that c-Rel is dispensable for mature Treg cell maintenance.

In contrast to animals with systemic c-Rel deficiency, mice with conditional deletion of c-Rel in Treg cells develop signs of inflammation with age, and c-Rel-deficient Treg cells are unable to control pathogenic T cells in a T cell transfer colitis model [26], demonstrating a role for c-Rel in Treg cell–mediated immune control. This was also observed in cell transfer experiments which showed that c-Rel knockout Treg cells are deficient in controlling the expansion of wild-type T cells in lymphopenic hosts [22]. Along the same line, conditional ablation of c-Rel in Treg cells affects a distinct set of genes (around 300 are up- and 350 downregulated) [26]. When focusing on CD44^hi^CD62L^lo^ effector-like or activated Treg cells, c-Rel ablation caused a pronounced loss of gene expression typical for in vivo activated Treg cells. Intriguingly, mice lacking c-Rel specifically in Treg cells display strongly enhanced anti-melanoma T cell responses, and this phenotype is dominant when c-Rel is globally inhibited by pentoxifylline [27]. Whether the selective effect of c-Rel deficiency on Treg cells with an activated phenotype (and not on Treg cells with a resting phenotype) indicates a preferential role of c-Rel for Treg cell function or for a specific Treg cell subset remains to be explored.

### RelA (p65)

Mice with a systemic knockout of RelA show embryonic lethality which can be rescued by the additional knockout of TNF [28]. Despite the only slightly reduced numbers of Treg cells in the thymus, the combined absence of RelA and TNF prevents the egress of Treg cells from the thymus to the periphery. Using fetal liver chimeras, the authors provide evidence that Treg cell-extrinsic functions of RelA/TNF in the radio-resistant thymic microenvironment are required for thymic egress [29]. Further studies revealed that the NF-κB subunit RelA has a central role in the maintenance of mature Treg cell identity and in the prevention of autoimmunity [26, 30]. Mice with specific deficiency for RelA in Treg cells develop a severe and early spontaneous systemic autoimmune syndrome that is associated with a defect of Treg cells [26, 30, 31]. These RelA-deficient Treg cells were shown to be unstable, lost FOXP3 expression, and produced inflammatory cytokines [30]. Chromatin immunoprecipitation followed by sequencing (ChIP–seq) revealed that RelA controls expression of genes linked to Treg cell identity as well as FOXP3-bound genes. Of note, RelA-controlled genes differ considerably between TCR-activated Treg and effector T cells, further strengthening the view that RelA dictates a Treg cell–specific gene signature [26]. Additionally, RelA deficiency results in an almost complete block in the generation of ROR(γt)^+^ Treg cells [31], suggesting an important role of RelA not only in thymic Treg cell differentiation but also in peripheral Treg cell differentiation. Whether RelA exerts this role by a related or different mechanism remains to be determined.

### RelA/c-Rel

Constitutive lack of both canonical NF-κB subunits in Treg cells leads to a phenotype very similar to that of scurfy mice, which lack Foxp3 expression and therefore Treg cells [26], confirming that canonical NF-κB is critical for Treg cell generation and/or maintenance. Inducible ablation of both transcription factors led to a reduction in Treg cell frequencies and dramatically affected signature gene expression, indicating that canonical NF-κB maintains Treg cell numbers and identity. Indeed, transcriptionally RelA/c-Rel double-deficient Treg cells clustered with wild-type conventional CD4 T cells. The canonical NF-κB-dependent genes only partially overlapped with Foxp3-dependent genes, demonstrating that these transcription factors control an independent set of Treg cell signature genes [26]. Induced individual ablation showed that c-Rel has a dominant role in Treg cell maintenance, CD25 and GITR expression as RelA ablation alone had no effect.

### p105/p50

The p50 subunit of NF-κB is not synthesized as an active DNA-binding protein, but is generated by proteolytic processing of the precursor p105, which contains p50 in its N-terminal half [32, 33]. The in vivo function of p105 in NF-κB regulation has been investigated using a knock-in approach, which involved the insertion of a stop codon in the processing site of p105, allowing the direct production of p50 without generating p105. p105 is dispensable for Treg cell development [34], but one report describes a role for p50 in Treg cell differentiation, which was suggested to occur through two distinct pathways [35]: Foxp3^−^CD25^hi^ Treg precursors reflect agonist selection, while Foxp3^lo^CD25^−^ Treg cell precursors undergo a process resembling normal positive selection. The latter differentiation path may be dependent on stimulation through CD28 and p50 signaling.

## Non-canonical NF-κB pathway components in Treg cell biology

Several studies have revealed important roles of the alternative NF-κB pathway (see Box 1) in Treg cell development and maintenance. The kinases NIK (MAP3K14) and IKKα both act upstream of NF-κB2 and RELB and are involved in the activation of non-canonical NF-κB signaling [36]. NIK links several co-stimulatory TNF receptor family members (TNFRs) to non-canonical NF-κB activation. These receptors include TNFR2, TNFRSF4 (CD134, OX40) [37], TNFRSF18 (GITR) [38], and TNFRSF9 (CD137, 4-1BB) [39], all of which were implicated in Treg cell function or phenotypic stability. Several studies demonstrate a critical function for NIK specifically in T cells [40–44]. NIK^−/−^ and NIK mutant *aly/aly* mice have reduced numbers of Treg cells. However, restoring NIK specifically in dendritic cells was shown to normalize thymic Treg cell numbers in NIK^aly/aly^ mice [45]. This finding suggests that NIK controls Treg cell numbers cell-extrinsically via dendritic cells. Further reports show a cell-intrinsic requirement for NIK in peripheral homeostasis of Treg cells [46, 47]. Constitutive enforced expression of NIK in all T cells impairs Treg cell function [48], while NIK over-expression restricted to Treg cells induced peripheral expansion of poorly functional Treg cells, resulting in an autoimmune pathology. Here, constitutive over-expression of NIK in Treg cells impaired their suppressive function and promoted pro-inflammatory cytokine production by Treg cells [49]. However, enforced expression of NIK can activate both alternative and canonical pathways [50], and therefore, the specific contribution of the alternative NF-κB pathway to these phenotypes remains to be elucidated.

Moreover, mice with conditional ablation of IKKα in T cells show a reduced frequency of thymic Treg cells as well as in peripheral lymphoid tissues but also a proliferative expansion of CD4^+^ T effector cells [51]. Whether these two observations depend on each other remains to be investigated through more targeted approaches. Since IKKα can also activate canonical NF-κB signaling [52], it has been suggested that the effects of NIK or IKKα rely more on impaired p65 or c-Rel activation rather than on diminished activation of the alternative NF-κB pathway. Thus, Treg cell alterations in the absence of these kinases might not reflect direct effects on non-canonical NF-κB signaling.

### Alternative NF-κB subunits in Treg cell biology

NF-κB2 and RelB are important regulators of immune tolerance, as shown by germline deletion of the respective proteins in *Relb*^*−/−*^ and *Nfkb2*^*−/−*^ mice, which results in spontaneous autoimmunity (reviewed in [53]). Mice lacking RelB display T cell–dependent inflammatory cell infiltration in several organs, myeloid hyperplasia, and splenomegaly due to extramedullary hematopoiesis [54]. Of note, alternative NF-κB signaling (again in crosstalk with canonical NF-κB) additionally plays a key role in the proper maturation and function of medullary thymic epithelia cells (mTECs), and deficits in this cell type directly and indirectly affect negative selection and thymic Treg cell differentiation [55].

Conditional inactivation of *Nfkb2* and/or *Relb* in both total T cells and Treg cells demonstrate a crucial function for p100/NF-κB2 but not RelB in Treg cell function. Mice with a systemic deficiency in *Nfkb2* showed normal thymic development of Treg cells, but enhanced frequencies of peripheral eTreg cells [56]. Young mice with a Treg cell–specific inactivation of *Nfkb2* did not display abnormal inflammation or autoimmunity [57]. With age, however, mice lacking Nfkb2 specifically in Treg cells develop massive colonic inflammation due to an impaired suppressive function of p100-deficient Treg cells, despite these mice harboring an expanded Treg cell population in their peripheral lymph nodes [57]. The colonic phenotype is probably due to aberrant expression of inflammatory cytokines by p100-deficient Treg cells which suggests that these numerically expanded p100-deficient Treg cells have impaired suppressive activity.

Mice with conditional inactivation of *Relb* in T cells or specifically in Treg cells did not show signs of autoimmunity and contained similar frequencies of Foxp3^+^ Treg cells in the periphery as wild-type controls [58]. In fact, inactivation of both *Relb* and *Nfkb2* reduced the inflammatory phenotype, demonstrating an essential role for p100 as an inhibitor of RelB in Treg cells [57]. Extrinsically, non-canonical NF-κB activity in dendritic cells may be a key factor that regulates the composition of the Treg cell compartment both in quantitative and qualitative terms [59].

## Atypical members of the IκB protein family and their role in Treg cell biology

The transcriptional activity of NF-κB is fine-regulated in the nucleus by a variety of mechanisms, including post-translational modifications of REL proteins as well as by atypical members of the NF-κB protein family. Classical IκBs are distinguished from the atypical IκB proteins of the Bcl-3 subfamily, including Bcl-3, IκBzeta (IκBζ, encoded by the *Nfkbiz* gene), and IκB_NS_ (encoded by the *Nfkbid* gene) [60]. The latter proteins are not degraded after NF-κB activation but are highly induced and act as transcriptional modulators with inductive and repressive capacities by binding to NF-κB transcription factors in the nucleus [61, 62]. Although these proteins formally belong to the IκBs due to the presence of ankyrin repeats in their structure, they do not functionally act as repressors of NF-κB nuclear localization but rather as modulators of NF-κB-mediated transcription. Atypical members can dramatically alter NF-κB-mediated transcription, both qualitatively and quantitatively, via the regulation of dimer exchange, the recruitment of histone-modifying enzymes, or the stabilization of NF-κB dimers bound to DNA [63].

### B cell leukemia 3 (Bcl-3)

Bcl-3, which was originally identified as a proto-oncogene in a subgroup of B cell leukemia patients, enters the nucleus and associates directly with DNA-bound NF-κB p50 or p52 homodimers to regulate NF-κB-dependent gene transcription. Conditional overexpression specifically in mouse T cells, the authors observed an impairment in the development of Th2, Th1, and Th17 cells in vivo [64]. Reissig et al. demonstrated that Bcl-3 is also important for the maintenance of Treg cell function and the prevention of spontaneous colitis. Here, the authors showed that in Treg cells, Bcl-3 interacts with p50 and inhibits p50 DNA binding and thereby alters the NF-κB-mediated genetic programs required for Treg cell development and function [65]. By contrast, using conditional knockout mice, it was further demonstrated that loss of Bcl-3 specifically in Treg cells was sufficient to boost RORγt^+^ Treg cell formation and resistance of mice to dextran sulfate sodium-induced colitis. Thus, Bcl-3 suppresses the accumulation of ROR(γt)+ Treg cells [66]. This report reveals a novel role for Bcl-3 in modulating NF-κB activation in Treg cell subset differentiation with possible clinical implications while the exact molecular mechanism still needs to be evaluated.

### IκB_NS_

The atypical inhibitor of NF-κB, IκB_NS_, also known as TA-NFKBH and Nfkbid, is the smallest member of the Bcl-3 subfamily [67]. IκB_NS_ can interact with several different NF-κB dimers in the nucleus and was shown to be involved in the development of Treg cells by regulating the transition of thymic immature GITR^+^CD25^+^Foxp3^−^ Treg cells into Foxp3^+^ mature Treg cells [68]. IκB_NS_ is transiently expressed during thymic nTreg cell development and drives Foxp3 induction by binding to its promoter and CNS3 via p50 and c-REL [68]. IκB_NS_ repression might ensure silencing of IL-2 transcription in Treg cells, as it is needed for IL-2 induction upon activation of CD4 and CD8 cells [69]. Although the protein is repressed in Foxp3^+^ Treg cells, IκB_NS_ is important for the maturation of Foxp3^−^ Treg cell precursors [68], as thymic Treg cells are reduced in IκBNS-deficient mice.

Interestingly, mice deficient for IκB_NS_ or c-REL show similar defects in the function of adaptive and innate immune cells, including a reduction of CD25^+^Foxp3^−^ Treg cell precursors. On the molecular level, c-REL and IκB_NS_ were reported to bind both to the core promoter and the CNS3 region of the *Foxp3* locus [70, 71]. In c-rel/IκB_NS_ double-deficient mice, Treg numbers are dramatically reduced, but these mice did not develop autoimmunity even when mice were aged more than 1 year, suggesting essential roles for c-REL and IκB_NS_ in T effector cell functions. Distinguishing the two proteins, it was shown that c-REL, but not IκB_NS_, controls the generation of classical CD25^+^Foxp3^−^ precursors via direct binding to the *Il2ra* locus [70]. In contrast to natural (n)Treg development in the thymus, similar to c-Rel, IκB_NS_ is dispensable for maintenance of mature Treg cells**.** This is supported by the fact that Nfkbid itself is suppressed in mature Treg cells [72].

### IkBζ

In vitro, IκBζ -deficient T cells have a high capacity for generating Treg cells when T cells are cultured under TGF-β stimulation in the presence of cytokine-neutralizing antibodies. Mechanistically, the authors propose that IκBζ itself negatively regulates the activation of the *Foxp3* promoter in a NF-κB-dependent manner [73]. The generation of Treg cell specific IκBζ-deficient mice revealed no deficiency in peripheral Treg cells in lymphoid organs. However, Treg cells from T cell–specific IκBζ-deficient mice were shown to have reduced immunomodulatory function [73]. Furthermore, IκBζ was shown to be induced by TGF-β signaling and plays a pivotal role in maintaining normal frequencies of Th17 cells. IkBζ binds, together with RORγ or RORα, to the IL-17a locus but whether a related pathway also affects Treg cells remains to be investigated [74].

## Insights into Treg cell differentiation from human patients

Analysis of patients with mutations in individual NF-κB members only infrequently directly assess Treg cells frequencies and functions. Interpretations of the resulting data are complicated due to the often-observed (severe) combined immune deficiency in these patients that can affect Treg cells indirectly. Some of the clinical manifestations in such patients resemble features that one may expect from mutations directly affecting Treg cell function, suggesting a possible role for NF-κB signaling for proper Treg development and function in humans.

Overall, there is strong evidence that canonical NF-κB signaling is critical for human Treg cell development. Severe combined immunodeficiency due to loss of IKK2 is characterized by a near-complete absence of Treg cells [75]. Similarly, nonsense mutations in IKBKB caused a paucity in Treg cell numbers in four infants along with combined immunodeficiency [76]. Analysis of patients with late-onset immune deficiency led to the identification of patients with heterozygous point mutations in IKK2 (IKK2V203I) that enhance NF-κB activation. In one patient, this was shown to correlate with increased Treg cell proportions in the peripheral blood and slightly increased FOXP3 protein levels. Engineering of the human mutation in mice essentially recapitulated the Treg cell phenotype [77]. Mutations in adaptor molecules downstream the TCR were linked to low Treg cell numbers. For instance, mutations in CARD11, an essential molecule for the correct formation of the CARD11 BCL10 MALT1 (CBM) complex and activation of canonical NF-κB signaling, can result in low Treg cell numbers [78, 79]. Along the same line, homozygous missense mutation in mucosa-associated lymphoid tissue lymphoma translocation 1 gene (MALT1) in patients were shown to impair NF-κB activation in lymphocytes and result in a strong reduction in Treg cells [80, 81]. Likewise, one case with an autosomal-recessive, complete BCL10 deficiency also showed a complete absence of Treg cells in the peripheral blood [82]. Collectively, the data imply a strong reliance of human Treg cell development on TCR-induced NF-κB activation, although signaling input from TNF family receptors can also play a role. This notion is further confirmed at the transcription factor level: patients with primary immune deficiency due to haploinsufficiency of NF-κB1/p105 have severely reduced Treg cell frequencies in their peripheral blood [31]. To what extent this reflects a preferential loss of Treg cells with high affinity TCRs as suggested from the mouse studies [35] remains to be investigated. Finally, the critical importance of c-Rel for Treg cell development was recently confirmed in a patient with inherited REL deficiency, who showed a reduction in Treg cell frequencies in the peripheral blood [83].

In the alternative branch of NF-κB signaling, homozygous loss-of-function mutations in NIK cause severe primary immune deficiency, but the proportions of Foxp3^+^ Treg cells were in the normal range in these patients [82]. Loss-of-function mutations due to the premature generation of a stop codon in the *RelB* gene in three consanguineous patients resulted in combined immune deficiency and overall lower T cell responsiveness [84]. Whether Treg cell numbers or function were also affected has not been reported due to the rapid stem cell transplantation for the treatment of these patients.

Altogether, there is strong evidence from monogenic human diseases that loss-of-function mutations either directly in NF-κB members or in adaptor molecules preventing proper canonical NF-κB activation strongly affect Treg cell development and/or maintenance. The roles of alternative NF-κB signaling in human Treg cells, by contrast, need to be clarified in future studies.

## Conclusions and perspectives

Evidence from mouse models clearly supports the notion that Treg cells require a certain degree of NF-κB activity to guarantee normal Treg differentiation. Loss-of-function studies involving IKK2, RelA, and c-Rel clearly demonstrated that canonical NF-κB activation is of particular importance for the differentiation, maintenance, identification, and function of thymic Treg cells. It should be noted that evidence from different genetic models, targeting either all T cells or specifically FOXP3^+^ T cells, warrants careful interpretation and cannot always be directly compared. While the first approach guarantees efficient knockout of NF-κB members already at an early state of Treg differentiation, the latter strategy is more targeted and excludes secondary effects due to the loss of the respective proteins in other T cell subpopulations possibly causing inflammation with subsequent effects on Treg cells. Treg cell–specific gene inactivation is restricted to later time points, when sufficient FOXP3 protein and Cre recombinase have been made. Also, in this experimental setting, the timing and extent of gene inactivation differ between individual *Foxp3*-Cre(ERT2)-transgenic mice, which can strongly influence the results. This is particularly relevant to study initial Treg cell differentiation during which NF-κB activity plays a pivotal role according to current knowledge (see above) because NF-κB may be relevant for ensuring Treg cell identity prior to Foxp3 expression [21]. In principle, recombination efficiencies and kinetics should be validated for each individual Cre-transgenic stain and the specific conditional allele employed [85]. The combination of single cell sequencing approaches and the use of novel mouse models along with CRISPR-Cas9 applications to manipulate Treg cells will allow in the future better insights into the individual or combinatorial role of NF-κB members for Treg cell biology.

Complete ablation of NF-κB proteins often allows a more direct comparison to mutations present in patients but often cannot answer whether Treg cell-extrinsic effects indirectly affect Treg cells. This is of high relevance for the design of T cell therapies that aim to restore Treg cells with adequate NF-κB activity. This basic NF-κB activity seems to be fundamental for Treg stability and identity and is thus of critical importance for such therapeutic approaches. Some NF-κB family members directly bind to regions known to regulate *Foxp3* gene expression. Stable FOXP3 expression ensured by NF-κB activity may exploit also a number of post-translational modification of the FOXP3 protein [86], but more research is needed for deeper mechanistic insights. Additionally, there is currently a fundamental lack of understanding whether and how NF-κB signaling affects the many versatile Treg cell subsets that were discovered in almost all tissues. These tissue-residing Treg cells often rely on a different number of co-transcription factors that ensure the tissue-specific function of Tregs and it will be important to investigate whether and how NF-κB will also affect these transcriptional regulators.

Another possibility to modulate NF-κB activity is to interfere with the signaling cascade that leads to NF-κB activation through the use of small molecules, but this strategy has additional caveats: First, one needs to identify a strategy to specifically target Treg cells and second, signaling upstream of NF-κB interferes with many other signaling cascades (e.g., cytokine receptor signaling) and may thus cause unexpected side effects. Currently, signaling via the TCR and its co-receptors is thought to be primarily responsible for NF-κB activation in Treg cells [9]. In addition, members of the TNF receptor superfamily were implicated in the generation of Tregs, in particular GITR, OX40 and TNFR2, all of which prominently activate NF-κB [87]. Thus, both homeostatic TCR signals and tissue-specific cytokines are necessary for Treg survival and this seems to depend on a basic NF-κB activity in Treg cells.

Activation of NF-κB and subsequent NF-κB-dependent gene activity is one of the basic principles of how innate immune cells respond to recognition of foreign danger-associated molecular patterns. This ancient principle was adapted by adaptive immune cells (both T and B cells). Why the same pathway is also necessary in Treg cells, which have a primarily immunosuppressive role, remains to be determined. One reason may be that controlling Treg activity by the same pathway that governs effector responses ensures a tightly controlled immune response and avoids overt tissue damage by overactivation of NF-κB pathways in other immune cells. Accordingly, the evolutionary conserved link of TCR activity to NF-κB activation is over-ruled by FOXP3 together with additional transcription factors to ensure the typical “immune-suppressive” signature in Treg cells over an NF-κB-induced “alert’ signature observed in other immune cells.

NF-κB transcription factors play diverse and important roles in both Treg cell differentiation and immune-modulating functions. Although great progress has been made in the past 10–15 years, many aspects of NF-κB functions in Treg cells remain unresolved. Determining the target genes, distinguishing common and unique roles of individual NF-κB members, and elucidating the Treg cell–specific gene regulation by NF-κB proteins remain important tasks for future studies. It will also be of particular importance to assess the role of NF-κB family members for the functionality of the diverse Treg cell subsets found in diverse peripheral organs and to distinguish between Treg cells differentiated in the thymus and Treg cells exclusively induced in the periphery. Altogether, both mouse and human data suggest that NF-κB family members hold substantial promise to modify Treg cell functions for the benefit of patients with unwanted Treg cell activity (e.g., cancer) or dysfunctional Treg cells (e.g., autoimmune diseases).

## Box 1 A brief introduction to NF-κB transcription factors

The core transcriptional mediators of the NF-κB family comprise a family of hetero- and homodimers composed of five related proteins, namely, p65 (RelA), c-Rel, RelB, p50 (NF-κB1), and p52 (NF-κB2), all comprising a conserved REL homology domain (RHD) near the N-terminus. This domain is crucial for dimer formation, binding to specific κB DNA sequences, nuclear import, and interaction with the inhibitors of kappa (IκB) family of regulatory molecules. In addition, the subunits RelA, RelB, and c-Rel harbor a C-terminal transactivation domain (TAD). Inactive NF-κB dimers localize within the cytoplasm, since the nuclear localization signal (NLS) within the RHD, is masked by IκBs [88].

Upon activation, NF-κB transcription factors are mobilized to the nucleus by a diverse range of stimuli that engage either the canonical (classical) or alternative (non-canonical) pathway. The canonical pathway activates dimers of RelA, c-Rel, and p50, whereas the nuclear translocation of p52–RelB dimers requires processing of p100–RelB heterodimers by the alternative pathway [88].

Different signal transduction networks engaged by extracellular signals all converge on an IκB kinase (IKK) complex comprising distinct kinase subunits, IKKα (IKK1), IKKβ (IKK2), and a regulatory protein, NEMO. In T cells, mainly the TCR and TNF receptor superfamily members activate the IKK complex by triggering phosphorylation of IKKβ, which in turn phosphorylates IκB proteins associated with NF-κB dimers. This initiates IκB polyubiquitination and degradation, which culminates in NF-κB nuclear translocation [88].

The alternative NF-κB pathway, engaged by ligands for TNF receptor superfamily members expressed in T cells, activates the IKK complex through NF-κB-inducing kinase (NIK) phosphorylation of IKKα. Activated IKKα in turn generates p52/p52 homodimers and p52/RelB heterodimers by triggering the proteolytic processing of p100 bound to p52 and RelB, respectively, in response to IKKα phosphorylation of p100. In resting cells, p100 sequesters RelB in the cytoplasm and signal-induced p100 processing allows for nuclear activation of RelB:p52 heterodimers [36]. However, p100 may retain RelA and c-Rel-containing heterodimers as well [89]. Interactions between canonical and non-canonical NF-κB were described in other cell types such as dendritic cells [90], but whether this interaction occurs also in Treg cells remains to be investigated. Finally, NF-κB dimers activated by both pathways regulate transcription by recognizing and binding to consensus κB elements located in the regulatory regions of target genes.

## Data Availability

Not applicable.
